# The RNA-binding domain of DCL3 is required for long-distance RNAi signaling

**DOI:** 10.1007/s42994-023-00124-6

**Published:** 2023-11-28

**Authors:** Jie Li, Bo-Sen Zhang, Hua-Wei Wu, Cheng-Lan Liu, Hui-Shan Guo, Jian-Hua Zhao

**Affiliations:** 1 State Key Laboratory of Plant Genomics, Institute of Microbiology, Chinese Academy of Sciences, Beijing, 100101 China; 2 CAS Center for Excellence in Biotic Interactions, University of the Chinese Academy of Sciences, Beijing, 100049 China; 3 Qilu Zhongke Academy of Modern Microbiology Technology, Jinan, 250022 China

**Keywords:** RNAi signaling, DCL3, RBD, sRNAs, *Cucumber mosaic virus*

## Abstract

Small RNA (sRNA)-mediated RNA silencing (also known as RNA interference, or RNAi) is a conserved mechanism in eukaryotes that includes RNA degradation, DNA methylation, heterochromatin formation and protein translation repression. In plants, sRNAs can move either cell-to-cell or systemically, thereby acting as mobile silencing signals to trigger noncell autonomous silencing. However, whether and what proteins are also involved in noncell autonomous silencing have not been elucidated. In this study, we utilized a previously reported inducible RNAi plant, *PDSi*, which can induce systemic silencing of the endogenous *PDS* gene, and we demonstrated that DCL3 is involved in systemic *PDS* silencing through its RNA binding activity. We confirmed that the C-terminus of DCL3, including the predicted RNA-binding domain, is capable of binding short RNAs. Mutations affecting RNA binding, but not processing activity, reduced systemic *PDS* silencing, indicating that DCL3 binding to RNAs is required for the induction of systemic silencing. Cucumber mosaic virus infection assays showed that the RNA-binding activity of DCL3 is required for antiviral RNAi in systemically noninoculated leaves. Our findings demonstrate that DCL3 acts as a signaling agent involved in noncell autonomous silencing and an antiviral effect in addition to its previously known function in the generation of 24-nucleotide sRNAs.

**Supplementary Information:**

The online version contains supplementary material available at 10.1007/s42994-023-00124-6.

## Introduction

RNA silencing (also known as RNA interference, or RNAi) is a nucleotide sequence-specific process that includes RNA degradation, DNA methylation, heterochromatin formation and protein translation repression (Baulcombe 2005; Chinnusamy and Zhu 2009; Guo et al. 2018). RNAi in most eukaryotes is mediated by small RNAs (sRNAs) of 21 to 24 nucleotides (nt) in length. In plants, cytoplasmic posttranscriptional gene silencing (PTGS) is induced by 21- and 22-nt sRNAs, which are the products of double-stranded RNAs (dsRNAs) processed by Dicer-like ribonucleases such as DCL4 and DCL2, and they achieve specificity through base pairing with targeted RNA sequences (Carmell and Hannon 2004; Gasciolli et al. 2005; Meister and Tuschl 2004).

In the nucleus, sRNAs direct the silencing machinery to interfere with transcription from homologous RNA at the transcriptional level (TGS). This process requires 24-nt sRNAs and long noncoding RNA transcripts for de novo DNA methylation, a process known as RNA-directed DNA methylation (RdDM) (Jackson et al. 2002; Matzke and Birchler 2005; Zilberman 2008). Long noncoding RNAs are produced by a plant-specific DNA-directed RNA polymerase, Pol V (Chinnusamy and Zhu 2009; Pikaard et al. 2008). Another plant-specific Pol IV-mediated transcribed precursor is processed by RNA-dependent RNA polymerase, RDR2, into dsRNA, which is diced by DCL3 to form 24-nt sRNAs (Blevins et al. 2015; Herr et al. 2005; Kanno et al. 2005; Matzke et al. 2009; Matzke and Birchler 2005; Onodera et al. 2005; Singh et al. 2019; Xie et al. 2004; Zhang et al. 2007). A 24-nt sRNA/AGO4/Pol V-dependent noncoding RNA effector complex directs the DNA methyltransferase DRM2-dependent de novo methylation of the target locus (Cao and Jacobsen 2002; Matzke et al. 2009). Pol IV, RDR2 and DCL3 thus mediate the spread of methylation during TGS (Daxinger et al. 2009; Kanno et al. 2008; Pagliarani and Gambino 2019).

Additionally, a noncell autonomous process allows RNA silencing to spread cell-to-cell and throughout the whole plant (Palauqui et al. 1997; Smith et al. 2007; Voinnet and Baulcombe 1997). It is noteworthy that sRNAs can move either cell-to-cell or systemically, thus acting as mobile silencing signals (Molnar et al. 2010; Pagliarani and Gambino 2019). In plants, sRNAs produced by the endogenous inverted-repeat (*IR*) locus or exogenous *IR* transgene (exo-*IR*) function as mobile signals and mediate both PTGS and RdDM/TGS of the *IR* transcripts and silencing of endogenous targets (Guo et al. 2003). Interestingly, the nuclear silencing pathway acts *in cis* to reinforce the TGS of the exo-*IR* silencer, leading to a reduction in exo-*IR* transcription and exo-*IR*-derived sRNAs and therefore, restricting the noncell autonomous silencing of the endogenous target gene (Dong et al. 2011). For instance, local chemically induced expression of exo-*IR* (e.g., exo-*Pdsi*) silencers in plants is not able to efficiently silence related endogenous coding genes (e.g., endo-*PDS*) in systemic leaves in which the *PDS*-silenced photobleaching phenotype was limited to areas near the veins (Dong et al. 2011; Guo et al. 2003; Liu et al. 2019). Mutations affecting nuclear proteins Pol V and DRD1, a chromatin remodeling protein, relieved exo-*Pdsi* self-silencing, resulting in higher levels of *Pdsi* transcripts and *Pdsi*-derived sRNAs, which increased the noncell autonomous silencing of endo-*PDS* (Dong et al. 2011). However, mutations affecting nuclear proteins Pol IV, RDR2 and DCL3 required for amplification of the 24-nt siRNAs did not increase the noncell autonomous silencing of endo-*PDS* (Dong et al. 2011). In the *dcl3* and *nrpd1* (mutation in the first and largest subunits NRPD1 of Pol IV) mutant backgrounds, reduced systemic silencing of endo-*PDS* was detected (Dong et al. 2011). This finding offers the possibility that, in addition to producing the 24-nt sRNAs, DCL3 and Pol IV may also play roles in either the translocation of the signal or signal detection in recipient cells. In addition to containing RNase III domains for processing sRNAs, the RNA binding domain (RBD) of Dicer/DCL proteins has been shown to contribute to Dicer/DCL binding, cleavage or subcellular localization (Banerjee and Barraud 2014; Doyle et al. 2013; Jinek and Doudna 2009; Nanduri 2000; Nicholson 2014; Ramos et al. 2000; Tian et al. 2004).

Here, we examined the effect of DCL3 on the translocation of silencing signals by using previously reported transgenic inducible *PDSi* plants that carry a chemical-inducible Cre/loxP (CLX) recombination system to trigger the transcription of exo-*Pdsi* to silence endo-*PDS* (Fig. S1A) (Guo et al. 2003). Complementation of *dcl3/PDSi* crossed progeny by plants with full-length DCL3 or a mutation in the RNase III domain that was deleterious to 24-nt production restored the systemic endo-*PDS* silencing and photobleaching phenotype. However, complementation of *dcl3/PDSi* with deletion in the predicted RBD at the C-terminus of the DCL3 protein did not restore the systemic endo-*PDS* silencing phenotype. In vitro detection of RNA binding affinity with the C-terminal fragment indicated that DCL3 possesses short RNA binding activity. Taken together, our data demonstrate that DCL3 acts as a signaling agent involved in noncell autonomous silencing in addition to its previously known function in the generation of 24-nucleotide sRNAs.

## Materials and methods

### Plant growth conditions and virus inoculation

In this study, the *Arabidopsis* plants were in the Col-0 background, and the inducible *PDSi* line 2 was described previously (Guo et al. 2003). The *dcl3-5* mutants were a gift from Marjori Matzke.

*Arabidopsis* seeds were germinated on MS medium containing 3% (W/V) sucrose and 0.8% (W/V) agar. After ten days, seedlings were transplanted into soil at 22 °C with a 16/8 h light/dark cycle. *N. benthamiana* plants were grew at 25 °C under a 16/8 h light/dark cycle. To induce *PDS* gene silencing at the germination-stage, seeds were germinated on inductive medium containing 2 μmol/L 17β-estradiol. For the induction of *PDS* gene silencing at the post-germination stage, one-week-old seedlings were transferred from MS medium to inductive medium for one week and then returned to MS medium.

For virus inoculation, fresh sap from cucumber mosaic virus (CMV, Shan-Dong isolate) infected *N. benthamiana* leaves was prepared. Two-week-old *Arabidopsis* plants were inoculated by mechanical rubbing. Photographs were taken at 14 days postinoculation (dpi).

### Cloning and plasmids

The ClonExpress II or ClonExpress MultiS kit (Vazyme, China) was used to construct the following plasmids by recombination cloning following the manufacturer’s instructions. All oligonucleotide primers used are listed in Table S1.

pBI121-6myc*-DCL3* was generated by cloning the coding sequence (NM_001161190.2, 4743 bp) of *DCL3* from Col-0 plants into the BamHI-linearized vector pBI121-Myc.

To generate pBI121-6myc-*DCL3*_*mRIII*_ and pBI121-6myc-*DCL3*_*ΔRBD*_ constructs, corresponding sequences were amplified from pBI121-6myc-*DCL3*, and the PCR products were cloned into the BamHI-linearized vector pBI121-Myc. For the plocex-*DCL3*-EGFP, plocex-*DCL3*_*mRIII*_-EGFP and plocex-*DCL3*_*ΔRBD*_ constructs, the *DCL3*, *DCL3*_*mRIII*_ and *DCL3*_*ΔRBD*_ sequences were cloned into the SpeI/BamHI-linear vector plocex-EGFP.

For generating translational fusion with glutathione S-transferase (GST) protein, the fragments (F1, F2, F3, F4 and F5) of *DCL3* were amplified from pBI121-6myc-*DCL3* and cloned into the BamHI/XhoI-linearized vector pGEX-4T-2.

### Plant transformation

A standard floral dip method (Clough et al. 1998) was used to perform plant transformations. The constructs for transformation, *pDCL3*-6myc*-DCL3*, *pDCL3*-6myc*-DCL3*_*mRIII*_ and *pDCL3*-6myc*-DCL3*_*ΔRBD*_ were transformed into the *Agrobacterium* strain EHA105, and then transformed into *dcl3-5/PDSi*. Positive transformants were screened on MS medium by kanamycin and hygromycin resistance.

### RNA extraction and RNA gel blot analysis

Four-week-old seedlings of each indicated genotype with or without inducer treatment were used for total RNA extraction, following the TRIzol reagent (Invitrogen) manufacturer’s instructions. 65 µg of total RNA was loaded for low-molecular-weight RNA blots, which were probed with biotin labeled specific oligonucleotide sequences (Table S1).

### qRT–PCR

To detect the relative accumulation of mRNAs, gDNA wiper mix (Vazyme) was used to remove residual genomic DNA. Reverse transcription was conducted by HiScript II qRT Super mix (Vazyme). Quantitative PCR was performed in a Bio-Rad CFX96 Real-Time system using ChamQ SYBR qPCR Master Mix (Vazyme). *Arabidopsis* AT4g33380 was used as an internal control, and transcript levels of the target genes were quantified relative to it. Three independent biological replicates and three technical replicates for each sample were performed. The specific primers are listed in Table S1.

### Purification of recombinant proteins and RNA binding activity detection

For expression of GST-tagged DCL3 fragments, recombinant plasmids were transformed into BL21 cells and induced with 0.8 mM isopropyl b-D-1-thiogalactopyranoside (Sigma–Aldrich) in Luria–Bertani medium at 16 °C overnight. Glutathione Sepharose 4B (GE Healthcare) was used to purify GST-tagged proteins following the instructions of manufacture.

For electrophoretic mobility shift (EMSA), synthesized RNA oligos were labeled with 0.3 mM biotin and 40 units of T4 RNA ligase (Pierce RNA 3’End Biotinylation Kit, 20,160) in 50 pmol quantities. Annealing and binding reactions were performed as described in our previous study (Duan et al. 2012). Then, biotin-labeled RNA was detected by chemiluminescence (Chemiluminescent Nucleic Acid Detection Module, Thermo, 89,880). For microscale thermophoresis (MST), purified proteins were labelled with fluorescent dye (Nano Temper Technologies, RED-NHS 2nd Generation) at room temperature for 30 min and then kept in MST buffer (50 mM Tris–HCl pH 7.4, 150 mM NaCl, 10 mM MgCl_2_ and 0.01% Tween 20). Fluorescently labelled proteins (100 nM) were incubated with various RNAs at room temperature for 5 min. The protein and RNA mixtures were loaded into standard capillary tubes (Nano Temper Technologies, MO-K022) and measured by a Monnolith NT.115 instrument (Nano Temper Technologies, 2 °C, 15% light-emitting diode power and 80% MST power). MO.Affinity Analysis (v.2.3) and GraphPad Prism 6.0 software were used to perform the data analysis.

All oligonucleotide primers used are listed in Table S2.

### Subcellular localization assays

To detect the subcellular localization of DCL3, DCL3_mRIII_ and DCL3_ΔRBD_ proteins, plocex-*DCL3*-EGFP, plocex-*DCL3*_*mRIII*_-EGFP and plocex-*DCL3*_*ΔRBD*_-EGFP constructs were separately transformed into the *Agrobacterium* strain (EHA105). A single colony was inoculated into 20 mL of selection medium (LB containing 50 mg/L kanamycin, 25 mg/L rifampicin, 10 mM MES, and 20 mM acetosyringone) and cultivated at 28 °C and 200 rpm for 16 h. *Agrobacterium* cells were harvested and resuspended in 10 mM MgCl_2_ buffer (containing 200 mM acetosyringone), and adjusted to an optical density at 600 nm of 1.0. The resuspended cultures were incubated at room temperature for 3 h and infiltrated into *N. benthamiana* leaves, which were covered overnight before and after infiltration.

To observe the subcellular localization, 100 ng/mL 49,6-diamidino-2-phenylindole (DAPI) was used to stain the nuclei for 10 min before confocal microscopy. Confocal fluorescence of GFP and DAPI were captured with Leica TCS SP8, and images were processed with Adobe Photoshop software (Adobe Systems).

## Results

### Reduced systemic *PDS* silencing phenotype in the *dcl3* mutant background

**Fig. 1:**
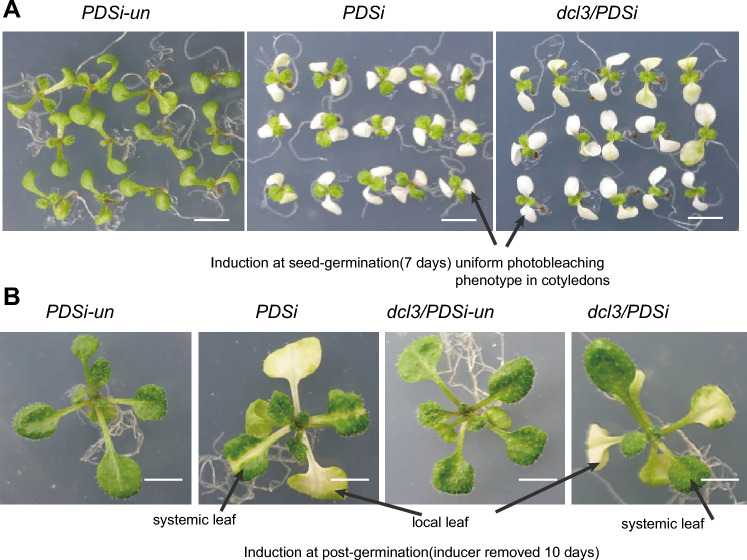
Reduction of the systemic *PDS* silencing phenotype in the *dcl3* mutant background.** A** Phenotypes of *PDS* silencing induced at the seed germination stage. A uniform photobleaching phenotype in cotyledons in *PDSi* and *dcl3/PDSi. PDSi*-un: without inducer treatment. Bar = 0.5 cm. **B** Phenotype of *PDS* silencing induced at the post-germination stage. No *PDS* silencing in systemic leaves in *dcl3/PDSi*. Photographs were taken 10 days after removing the inducer. Bar = 0.5 cm

We previously obtained a reduced photobleaching phenotype in crossed progeny *dcl3-1*/*PDSi* in the T-DNA insertion *dcl3-1* mutation background (SALK_005512) (Dong et al. 2011). In this study, we first verified the effect of *DCL3* in the *PDSi* system by using a point mutation, *dcl3-5* (Fig. S1B), to cross with the stable and homozygous *PDSi* line (line-2) (Guo et al. 2003). The resulting *dcl3-5/PDSi* (simplified as *dcl3/PDSi*) F1 progeny was self-fertilized to generate a segregating F2 population. Homozygous F3 seeds germinated on inductive medium, and all seedlings showed a uniform photobleaching phenotype in cotyledons similar to *PDSi* line-2 seedlings at 7 days (Fig. 1A). At the post-germination stage, *dcl3/PDSi* displayed no photobleaching in systemic leaves, while photobleaching limited to areas near the veins in systemic leaves was observed in *PDSi* seedlings (Fig. 1B), consistent with the previous result for *dcl3-1*/*PDSi* (Dong et al. 2011).

**Fig. 2:**
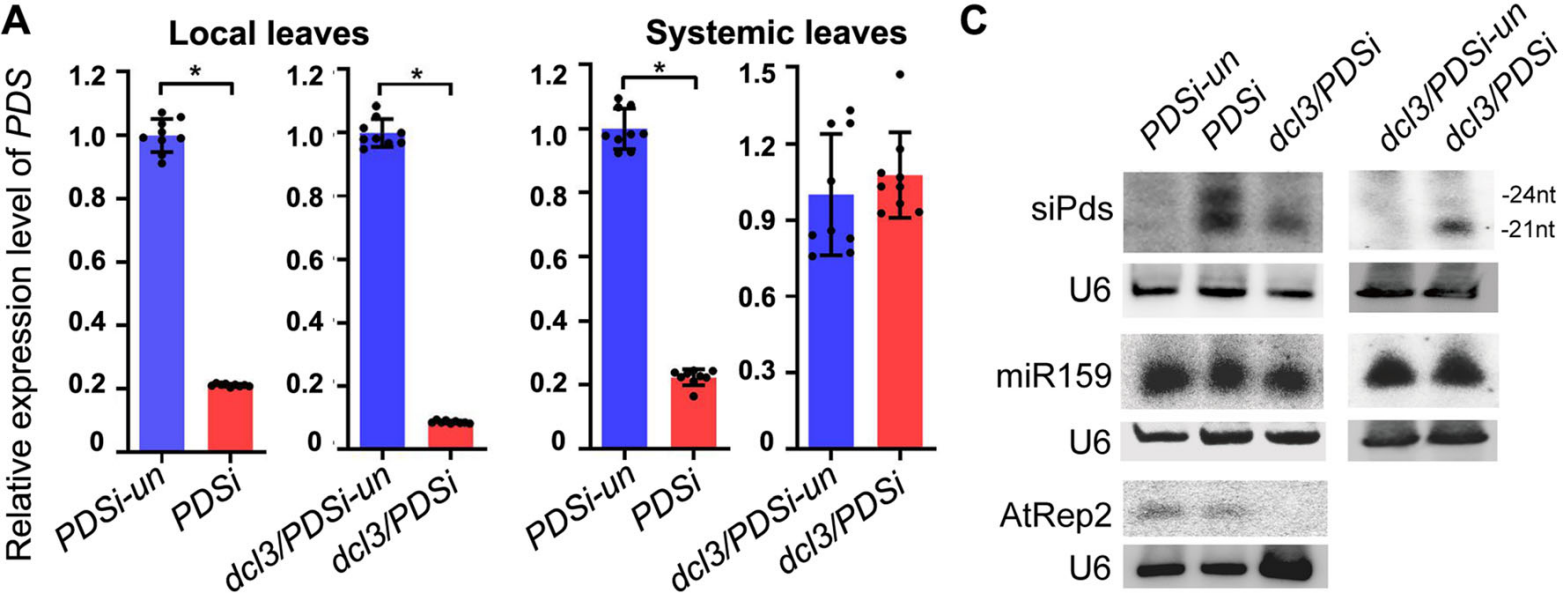
Detection of *PDS* mRNA and the accumulation of *PDS*-derived siRNAs (siPds) and endogenous sRNAs. **A**, **B** The relative expression of *PDS* in local (**A**) and systemic (**B**) leaves of various plants. Values are the means ± SD, and asterisks indicate statistically significant differences (*n* = 9, *t* test, *P* < 0.05). **C** Detected of siPds, AtRep2 and miR159 by RNA gel blotting. Biotin-labeled sense *PDS* RNA probes or oligodeoxynucleotide probes specific for AtRep2 and miR159 were used. U6 RNA hybridization was used as a loading control

The accumulation of *PDS* mRNA was examined in locally bleached leaves. *PDS* mRNA was reduced in locally silenced leaves of *PDSi* and *dcl3/PDSi* seedlings but was not reduced in seedlings without inducer treatment *PDSi* (*PDSi-un*) and *dcl3/PDSi* (*dcl3/PDSi*-*un*) (Fig. 2A). Samples of areas near the veins from systemic leaves were also collected for *PDS* mRNA detection. As expected, reduced accumulation of *PDS* mRNA was detected for *PDSi* but not *dcl3/PDSi* seedlings compared to that of *PDSi*-un and *dcl3/PDSi*-un (Fig. 2B). Accumulation of 21- and 24-nt siRNAs corresponding to the Pdsi region (designated as Pdsi-related, siPds) was found in *PDSi*-silenced samples, whereas only 21-nt siPds were detected in *dcl3/PDSi*-silenced samples (Fig. 2C). Accordingly, endogenous 24-nt siRNA, AtRep2, was absent in *dcl3/PDSi* mutant progeny, but miR159 was detected at a similar level in samples of all genotypes (Fig. 2C), verifying the DCL3 mutant allele and validating that DCL3 is required for 24-nt siRNA biogenesis and for noncell autonomous silencing, and *dcl3* mutation does not greatly affect the induction of local silencing.

### RNA binding activity of DCL3 is required for the induction of systemic silencing

**Fig. 3:**
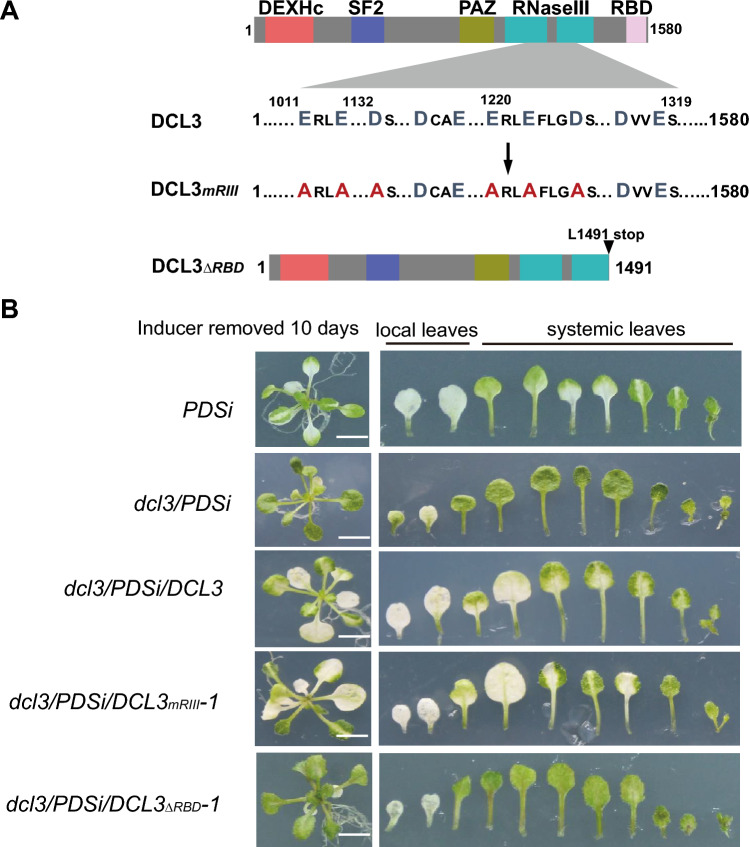
Analysis of the photobleaching phenotype in DCL3 and its derivative mutants. **A** Schematic of the DCL3 RNaseIII-deficient mutation, *DCL3*_*mRIII*_*,* and RBD-deletion mutation, *DCl3*_*∆RBD*_. **B** Phenotypes of *PDS* silencing induced at the post-germination stage in *PDSi*, *dcl3/PDSi*, *dcl3/PDSi/DCL3*, *dcl3/PDSi/DCL3*_*mRIII*_*-1* and *dcl3/PDSi/DCl3*_*∆RBD*_*-1*. Bar = 0.5 cm

According to the canonical RdDM pathway, DCL3 is responsible for cleaving dsRNA into 24-nt siRNAs (Wang et al. 2021; Zhang et al. 2022). We next examined whether RNase III activity was required for the induction of systemic silencing of endo-*PDS*. We first analyzed the DCL3 sequence and noticed that, in addition to the PAZ and RNase III domains required for processing sRNAs, DCL3 contains a potential RBD (Fig. 3A). We created complementation for *dcl3/PDSi* plants with a myc-tagged full-length *DCL3* sequence, a point mutation, *DCL3*_*mRIII*_, with six key residues of RNase III replaced by alanine (Fig. 3A), or a deletion mutant, *DCL3*_*ΔRBD*_, with deletion of the C-terminus of 89 amino acids, including the RBD domain and its upstream region (Fig. 3A), driven by the DCL3 native promoter. At the post-germination stage, the induced systemic silencing of endo-*PDS*, which limited photobleaching to areas near the veins in upper untreated leaves as in *PDSi* plants (Fig. 1B and S2A), was observed for complementary plants of *dcl3/PDSi/DCL3* and *dcl3/PDSi/DCL3*_*mRIII*_ but not *dcl3/PDSi/DCL3*_*ΔRBD*_ (Fig. 3B and Fig. S2A). Seeds of all complementary lines germinated on inductive medium showed a uniform photobleaching phenotype in cotyledons similar to *PDSi* and *dcl3/PDSi* seedlings at 7 days (Fig. S2B), indicating that the chemically inducible CLX recombination silencing system worked well upon induction in the transgenic plants. Therefore, our data suggest that its role in binding to RNAs but not the processing of sRNAs of DCL3 is required for the induced systemic silencing of endo-*PDS*.

**Fig. 4:**
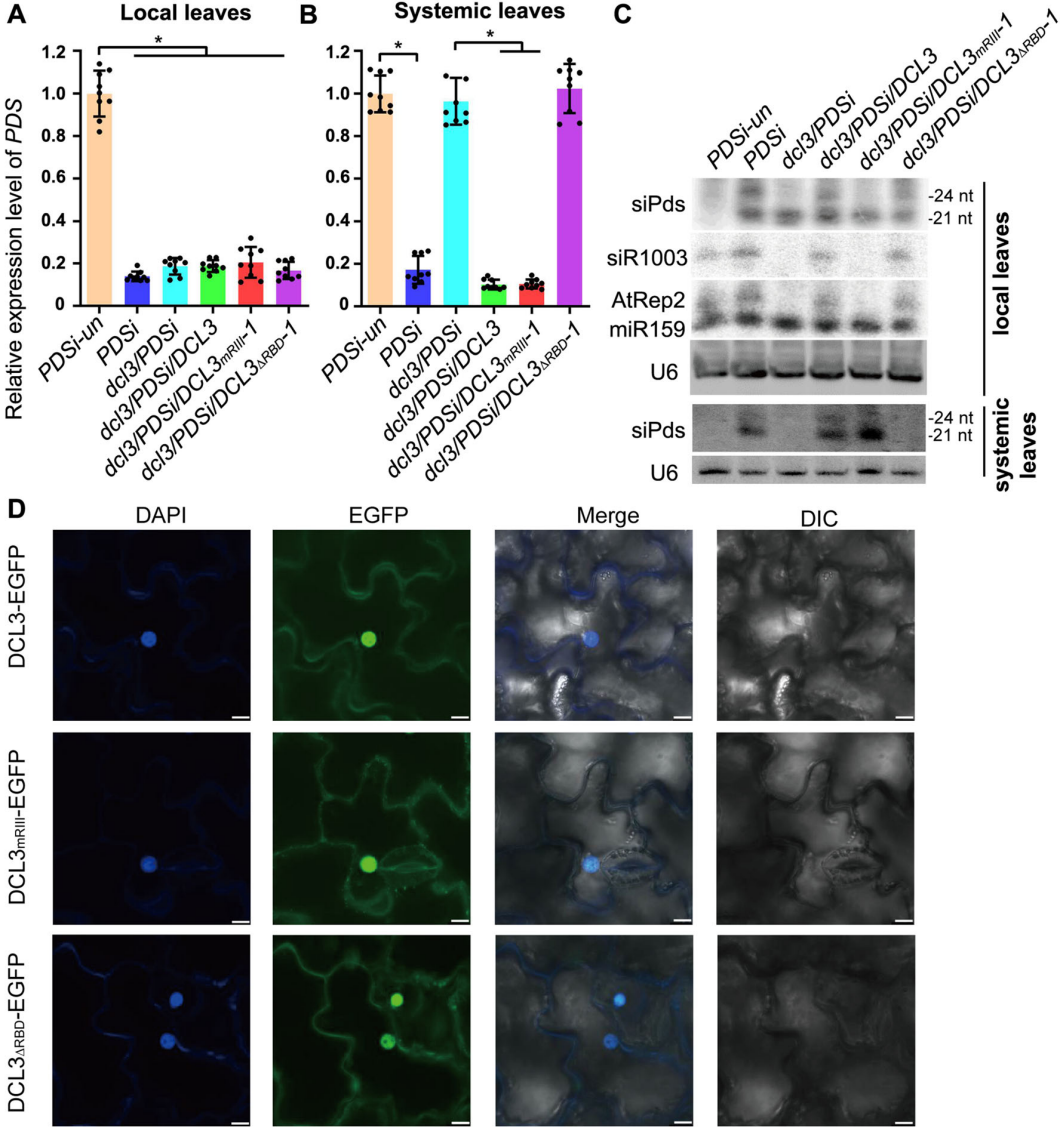
Analysis of *PDS* silencing in DCL3 and its derivative mutants. **A**, **B** Detection of *PDS* mRNA in local (A) and systemic (B) leaf samples. Values are the means ± SD, and asterisks indicate statistically significant differences (*n* = 9, one-way ANOVA, *P* < 0.05). **C** The accumulation of *PDS*-derived siRNAs (siPds), endogenous AtRep2, siR1003 and miR159 (RNA gel blotting). Biotin-labeled sense *PDS* RNA probes or oligodeoxynucleotide probes specific for endogenous sRNAs were used. Hybridization for AtRep2 and miR159 was performed with two probes mixed. U6 RNA hybridization was used as a loading control. **D** Subcellular localization of DCL3, DCL3_mRIII_ and DCL3_∆RBD_. Pairwise presentation of confocal microscopic images showing DAPI fluorescence and EGFP fluorescence in *N. benthamiana* expressing the indicated EGFP fusion proteins. One of the typical cells from each assay for confocal microscopy analysis is presented. Bar = 10 μm

The expression of DCL3, DCL3_mRIII_ and DCL3_ΔRBD_ in the transgenic complementation lines *dcl3/PDSi/DCL3, dcl3/PDSi/DCL3*_*mRIII*_, and *dcl3/PDSi/DCL3*_*ΔRBD*_ was confirmed by Western blot analysis (Fig. S3). Compared to the *PDSi-un* plants, the levels of *PDS* transcripts were significantly reduced in local leaves of *PDSi*, *dcl3/PDSi*, *dcl3/PDSi/DCL3*, *dcl3/PDSi/DCL3*_*mRIII*_ and *dcl3/PDSi/DCL3*_*ΔRBD*_ plants (Fig. 4A). Consistent with the photobleaching phenotype in systemic leaves, decreased *PDS* was detected only in *PDSi*, *dcl3/PDSi/DCL3*, and *dcl3/PDSi/DCL3*_*mRIII*_ but not in *dcl3/PDSi* and *dcl3/PDSi/DCL3*_*ΔRBD*_ plants (Fig. 4B). Northern blot analysis of siPds in local photobleaching leaves confirmed that 21- and 24-nt siPds were produced in *PDSi*, *dcl3/PDSi/DCL3* and *dcl3/PDSi/DCL3*_*ΔRBD*_ (Fig. 4C), but only 21-nt siPds were detected in *dcl3/PDSi* and *dcl3/PDSi/DCL3*_*mRIII*_. miR159 was detected at a similar level in samples of all genotypes (Fig. 4C). The finding that *dcl3/PDSi/DCL3*_*mRIII*_ plants lacked the 24-nt siPds capable of inducing systemic silencing of endo-*PDS* indicated that 24-nt sRNAs are not necessary for the induction of systemic silencing. Notably, many 21- and 24-nt siPds, as well as 24-nt AtRep2 and siR1003 (Fig. 4C), observed in seedlings of *dcl3/PDSi/DCL3*_*ΔRBD*_ with bleaching in local leaves failed to induce systemic silencing of endo-*PDS* (Figs. 3B and 4A). The 21- and 24-nt siPds were undetectable in systemic leaves of *dcl3/PDSi/DCL3*_*ΔRBD*_ plants (Fig. 4C), in agreement with the observation of comparable *PDS* transcripts in *dcl3/PDSi/DCL3*_*ΔRBD*_ and *PDSi-un* plants (Fig. 4B).

Next, we examined the subcellular localization of DCL3_mRIII_ and DCL3_ΔRBD_. DCL3, DCL3_mRIII_ and DCL3_ΔRBD_ fused with EGFP were transiently expressed in *N. benthamiana*. In agreement with a previous study (Hooper et al. 2017), the green fluorescence of EGFP from the DCL3-EGFP construct was mainly detected in the nucleus (Fig. 4D). We found that RNase III domain mutation and RBD domain deletion did not alter DCL3 nuclear subcellular localization (Fig. 4D). Taken together, our data demonstrate that the RNA binding activity of DCL3 rather than its sRNA processing activity plays an important role in noncell autonomous silencing.

### The C-terminal domain of DCL3 exhibits RNA binding activity

**Fig. 5:**
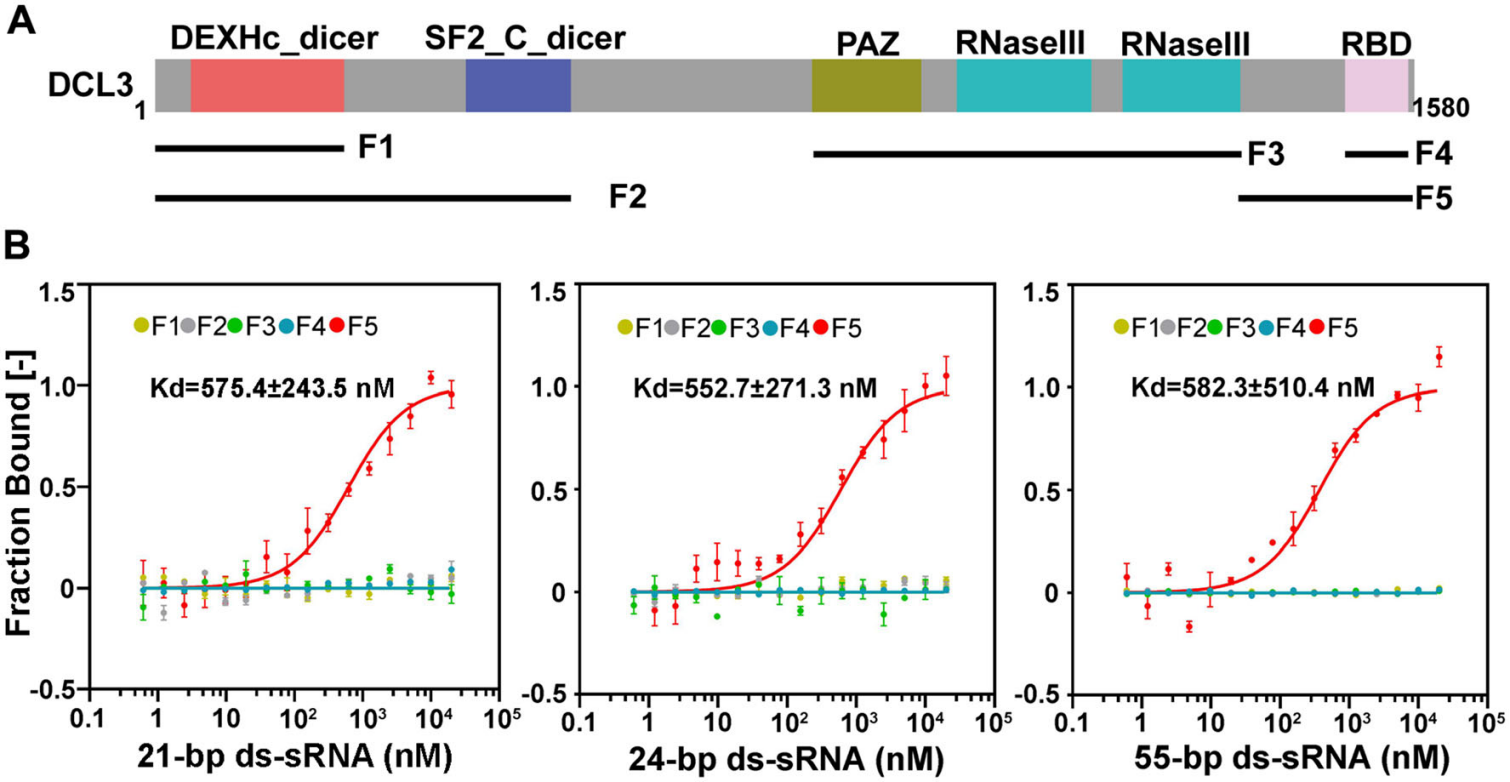
Detection of the RNA binding affinity of DCL3 by MST assay. **A** Schematic of the various GST-tagged fragments of DCL3 (F1, F2, F3, F4 and F5). **B** MST assay for detection of sRNA binding affinity of DCL3. Various GST-tagged fragments of DCL3 (as shown in A) were incubated with synthetic 21-, 24- and 55-bp (base pair) ds-sRNA. 100 nmol of purified proteins was loaded in each assay

We then characterized the RNA binding activity of DCL3 using EMSA and MST assays. Different fragments of DCL3, including F1, F2, F3, F4 and F5 (Fig. 5A), were expressed and purified as GST fusion proteins. For EMSA assay, GST and GST-tagged 2b, a CMV suppressor of RNA silencing (Duan et al. 2012), were used as negative and positive controls, respectively. Consistent with previous reports, GST-2b efficiently binds to the 21-bp (base pair), 24-bp and 55-bp ds-sRNA (Duan et al. 2012). EMSA and MST results showed that F5, containing the RBD domain and its upstream region, exhibited high affinity for ds-sRNAs (Fig. 5B and Fig S4A). Except for F5, none of the other fragments were capable of binding to ds-sRNAs (Fig. 5B and Fig S4A). The affinity of to various ds-sRNAs was quantified via MST yielding identical Kds, 575.4 ± 243.5 nM for 21-bp ds-sRNA, 552.7 ± 271.3 nM for 24-bp ds-sRNA and 582.3 ± 510.4 nM for 55-bp ds-sRNA (Fig. 5B).

**Fig. 6:**
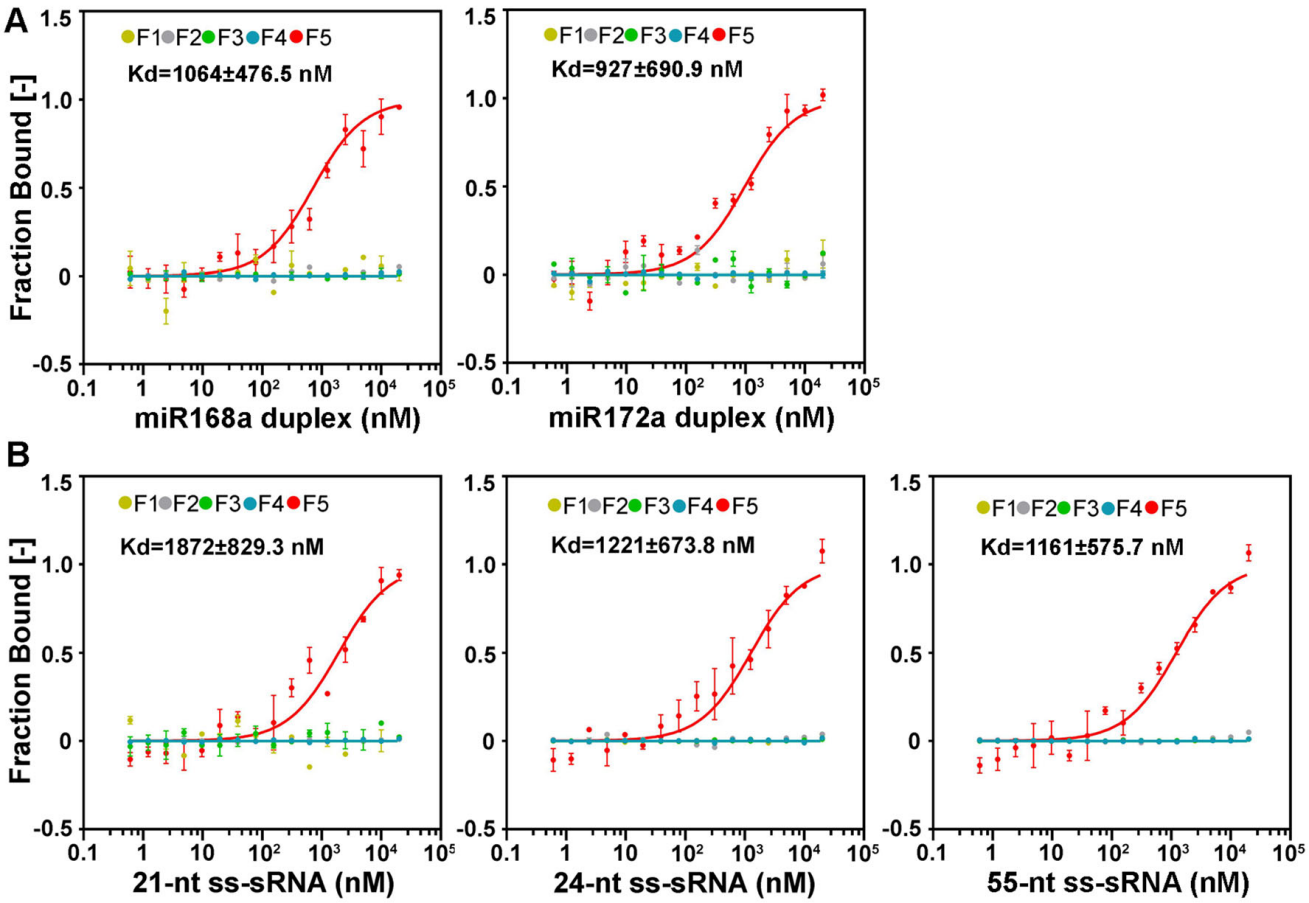
Detection of the miRNA duplexes and single-stranded siRNA binding affinity of DCL3. **A** GST-tagged F1, F2, F3, F4 and F5 were incubated with synthetic miRNA duplexes. **B** GST-tagged F1, F2, F3, F4 and F5 were incubated with 21-, 24- and 55-nt ss-sRNA. 100 nmol of purified proteins was loaded in each assay

Similar to the 2b protein, F5 also exhibited high affinity for sRNA duplexes formed between miRNA and miRNA*, yielding Kd of 1064 ± 476.5 nM for miR168a duplex and 927 ± 690.9 nM for miR172a duplex (Fig. 6A and Fig. S4B). However, unlike the 2b protein, F5 is capable of binding to single-stranded (ss)-sRNAs of 21 (Kd = 1872 ± 829.3 nM), 24 (Kd = 1221 ± 673.8 nM), and 55-nt (Kd = 1161 ± 575.7 nM) in length (Fig. 6B and Fig. S4C). Our results demonstrate that the RBD and its upstream region contribute to the RNA binding activity of DCL3, and the predicted RBD domain per se was inactive in binding to RNAs. Taken together, our data verified that DCL3 possesses RNA binding activity and efficiently binds to double- or single-stranded 21-bp sRNAs, 24-bp sRNAs, and 55-bp sRNAs.

### RNA binding activity of DCL3 is required for systemic antiviral silencing

A previous study showed that DCL3-dependent 24-nt sRNAs were inactive in targeting homologous viral RNAs for degradation, but loss of DCL3 activity increased virus accumulation in *dcl2/dcl4* mutant plants (Ding and Voinnet 2007). Therefore, we finally examined whether the RNA-binding function of DCL3 was required for antiviral RNAi.

**Fig. 7:**
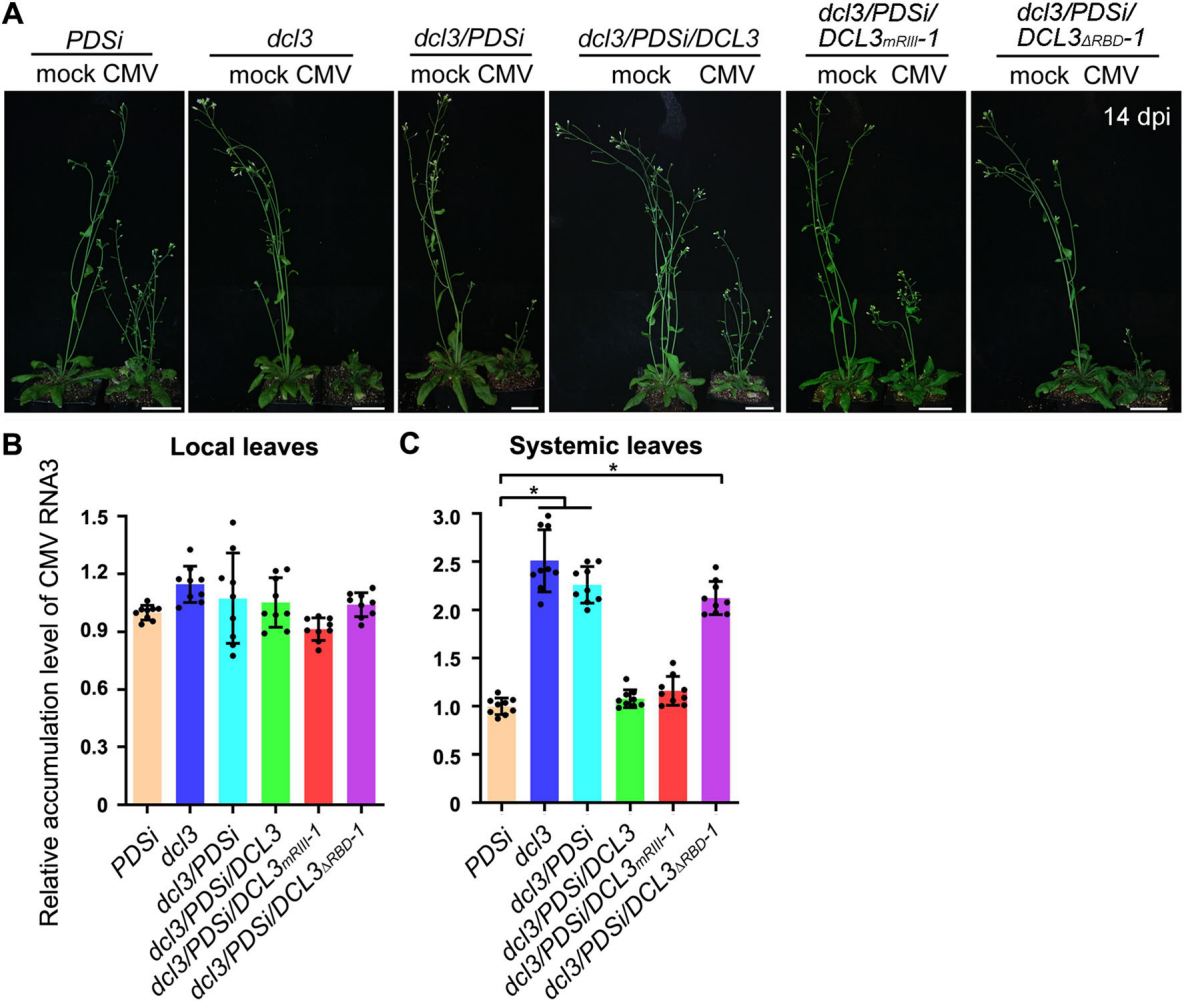
Analysis of CMV infection phenotypes and CMV RNA accumulation. **A** CMV disease symptoms in *PDSi*, *dcl3*, *dcl3/PDSi*, *dcl3/PDSi/DCL3*, *dcl3/PDSi/DCL3*_*mRIII*_ and *dcl3/PDSi/DCl3*_*∆RBD*_. Photographs were taken at 14 dpi. Bar = 5 cm. **B**, **C** Detection of CMV RNA accumulation by qRT–PCR. Total RNA was extracted from mock-inoculated, CMV-inoculated local leaves (**B**) and systemically infected leaves (**C**) at 11 dpi. Values are the means ± SD, and asterisks indicate statistically significant differences (*n* = 9, one-way ANOVA, *P* < 0.05)

Various genotypes of *Arabidopsis* transgenic plants were inoculated with CMV, and disease symptoms were observed. As shown in Fig. 7A, CMV-infected *PDSi*, *dcl3/PDSi/DCL3* and *dcl3/PDSi/DCL3*_*mRIII*_ exhibited similar developmental defect infectious disease symptoms, and CMV-infected *dcl3*, *dcl3/PDSi* and *dcl3/PDSi/DCL3*_*ΔRBD*_ displayed more developmental defect-related infectious symptoms, with more fasciated new leaves and defective inflorescence (Fig. 7A). Consistent with the disease symptoms, qRT–PCR detected higher viral RNA accumulation in systemic leaves of CMV-infected *dcl3*, *dcl3/PDSi* and *dcl3/PDSi/DCL3*_*ΔRBD*_ compared to that of *PDSi*, *dcl3/PDSi/DCL3* and *dcl3/PDSi/DCL3*_*mRIII*_ plants, while accumulation of viral RNAs reached similar levels in locally inoculated leaves in all plant genotypes (Fig. 7B and C). Taken together, our results demonstrate that DCL3 plays a role in systemic anti-CMV silencing, which requires its RNA-binding function to translocate viral sRNAs produced in locally inoculated leaves to noninoculated systemic leaves before or during viral systemic movement.

## Discussion

The chemically inducible Cre/loxP recombination system triggers the expression of exo-*Pdsi* RNA to induce endo-*PDS* silencing, providing a useful system to study the signaling mechanisms of RNAi in plants without the need for grafting (Guo et al. 2003). The merits of silencing endogenous *PDS* mRNA are the visible photobleaching phenotype and the ability to distinguish local and systemically silenced leaves (Dong et al. 2011; Guo et al. 2003; Liu et al. 2019). Given the existence of negative autoregulation of the exo-*Pdsi* through TGS, which acts in *cis* to reinforce self-silencing of the exo-*Pdsi* and results in reduced production of siPds and restrains the noncell autonomous silencing of endo-*PDS*, the *PDS*-silenced photobleaching phenotype in systemic leaves was limited to areas near the veins, distinct from the local *PDS*-silenced leaves with uniform photobleaching (Dong et al. 2011; Guo et al. 2003; Liu et al. 2019). This provides a convenient means to create genetic materials for dissection of RNAi signaling mechanisms.

In this work, by crossing *PDSi* plants with plants containing a point mutation, *dcl3-5*, the *dcl3/PDSi* progeny did not exhibit the photobleaching phenotype in the upper leaves upon chemical induction at the post-germination stage, indicating failure to induce systemic silencing of endo-*PDS* in the absence of *DCL3*. DCL3 mainly localizes in the plant nucleus and is well known for its production of 24-nt sRNAs in the nuclear RNAi pathway (Mari-Ordonez et al. 2013). However, the failure to induce endo-*PDS* silencing in systemic leaves was not due to the lack of 24-nt siPds in the *dcl3/PDSi* plants because the *dcl3/PDSi/DCL3*_*ΔRBD*_ complementation plants with high accumulation of 21- and 24-nt siPds failed to induce systemic silencing of endo-*PDS*, despite good induction of local *PDS* silencing (Fig. 4C and S2B). In contrast, *dcl3/PDSi/DCL3*_*mRIII*_ complementation plants without production of 24-nt siPds showed strong induction of systemic endo-*PDS* silencing, similar to the *dcl3/PDSi/DCL3* complementation plants (Fig. 4C and S2B).

In addition to mobile sRNAs, proteins that are capable of binding sRNAs possibly act as signaling agents. The detection of a single-stranded small (ss)-sRNA-binding protein (PSRP1) in phloem sap from cucumber and lupin suggests that ss-sRNA is selected by PSRP1 for transport through the vascular transport system (Yoo et al. 2004). In this study, we found that *d*c*l3/PDSi/DCL3*_*mRIII*_ but not *dcl3/PDSi/DCL3*_*ΔRBD*_ plants were capable of inducing systemic silencing of endo-*PDS*. Complementation of *d*c*l3/PDSi* plants with the *DCL3*_*mRIII*_ mutant gene retaining the RBD-containing C-terminal domain restored the ability to induce endo-*PDS* silencing in systemic leaves, and our data demonstrate that DCL3 binding to RNAs rather than its sRNA processing activity is required for RNA silencing-mediated signaling. In view of the capability of DCL3 to bind RNAs, we cannot rule out the possibility that sRNA precursors bound by DCL3 are involved in RNA silencing signaling.

Double-stranded RNA binding (DRB) proteins are considered promoting cofactors of DCL proteins during the precise production of sRNAs (Montavon et al. 2017). However, unlike other DCL proteins, such as DCL1 and DCL4, which require DRBs for proper production of sRNAs, DCL3 does not require any DRB to efficiently generate 24-nt sRNAs (Montavon et al. 2017). Interestingly, we found that complementation of *d*c*l3/PDSi* plants with *DCL3*_*ΔRBD*_, lacking a C-terminal RBD domain, is capable of producing 24-nt siPds, demonstrating that RBD activity is not required for generation of 24-nt sRNAs. Unlike PSRP1, which selectively binds to ss-sRNAs (Montavon et al. 2017), or CMV-2b, which only binds to ds-sRNAs, the RBD-containing C-terminal fragment of DCL3 binds to ds-sRNAs or ss-sRNAs. Complementation of *dcl3/PDSi* plants with RBD-containing DCL3 restored the induction of systemic *PDS* silencing. Therefore, DCL3 binding to RNAs is unlikely to astrict but assists their cell-to-cell or long-distance movement. Complementation of *dcl3/PDSi* plants with RBD-containing DCL3 also restored systemic anti-CMV silencing, suggesting that the single- and double-stranded RNA binding activity of DCL3 was superior to CMV-2b’s double-stranded RNA binding activity in the suppression of antiviral silencing. Nevertheless, there is an interesting assumption that RBD activity of DCL3 is required for virus movement, which is worth testing. Previous studies in human and yeast Dicers have shown that RBDs are critical for nuclear localization of the Dicer proteins (Banerjee and Barraud 2014; Doyle et al. 2013). Unlike mammalian Dicers, which predominantly localize to the cytoplasm, plant DCL3 mainly localizes in the nucleus, and we found that the RBD of DCL3 is not required for DCL3 nuclear localization. Together with the vascular expression of DCL3 (http://bar.utoronto.ca/eplant/), our data demonstrate that DCL3 acts as an RNA silencing signaling agent for mobile sRNAs to trigger noncell autonomous systemic silencing, in addition to its previously known function in the generation of 24-nucleotide sRNAs for de novo DNA methylation. However, the precise mechanism by which DCL3 triggers systemic silencing needs to be further elucidated. Whether DCL3 has the capacity to move from cell to cell through plasmodesmata remains to be examined. In addition, whether and how DCL3 carrying sRNAs can enter the vascular system for long-distance movement are poorly understood.

### Supplementary Information

Below is the link to the electronic supplementary material.

Supplementary file1 (DOCX 2824 KB)

## Data Availability

All data generated or analyzed during this study are included in the manuscript and its Supplementary file.
