# Publisher Correction: The RNA-binding domain of DCL3 is required for long-distance RNAi signaling

**DOI:** 10.1007/s42994-023-00128-2

**Published:** 2023-12-15

**Authors:** Jie Li, Bo-Sen Zhang, Hua-Wei Wu, Cheng-Lan Liu, Hui-Shan Guo, Jian-Hua Zhao

**Affiliations:** 1 State Key Laboratory of Plant Genomics, Institute of Microbiology, Chinese Academy of Sciences, Beijing, 100101 China; 2 CAS Center for Excellence in Biotic Interactions, University of the Chinese Academy of Sciences, Beijing, 100049 China; 3 Qilu Zhongke Academy of Modern Microbiology Technology, Jinan, 250022 China

**Correction to: aBIOTECH** 10.1007/s42994-023-00124-6

The copyright holder for this article was incorrectly given as 'Agricultural Information Institute, Chinese Academy of Agricultural Sciences' but should have been 'The Authors'.

This article was originally published with the incorrect licence; it should have been:

**Open Access** This article is licensed under a Creative Commons Attribution 4.0 International License, which permits use, sharing, adaptation, distribution and reproduction in any medium or format, as long as you give appropriate credit to the original author(s) and the source, provide a link to the Creative Commons licence, and indicate if changes were made.

The images or other third party material in this article are included in the article’s Creative Commons licence, unless indicated otherwise in a credit line to the material. If material is not included in the article’s Creative Commons licence and your intended use is not permitted by statutory regulation or exceeds the permitted use, you will need to obtain permission directly from the copyright holder.

To view a copy of this licence, visit http://creativecommons.org/licenses/by/4.0/.

