# Supplementary material for: The RNA-binding domain of DCL3 is required for long-distance RNAi signaling
Source: aBIOTECH. 2023 Nov 28;5(1):17–28. doi: 10.1007/s42994-023-00124-6 (PMC10987413; doi:10.1007/s42994-023-00124-6)
Supplement: Supplementary file 1 — Supplementary file1 (DOCX 2824 KB) [file 42994_2023_124_MOESM1_ESM.docx]

**Supplementary Information**

**The RNA-binding domain of DCL3 is required for long-distance RNAi signaling**

**Running title: DCL3 mediates sRNA long-distance transmission**

Jie Li^1,2^, Bo-Sen Zhang^1,2^, Hua-Wei Wu^1,2^, Cheng-Lan Liu^3^, Hui-Shan Guo^1,2^ and Jian-Hua Zhao^1,2*^

^1^State Key Laboratory of Plant Genomics, Institute of Microbiology, Chinese Academy of Sciences, Beijing 100101, China

^2^CAS Center for Excellence in Biotic Interactions, University of the Chinese Academy of Sciences, Beijing 100049, China

^3^Qilu Zhongke Academy of Modern Microbiology Technology, Jinan 250022, China

*Corresponding author: Jian-Hua Zhao

Email: [zhao_jian_hua@hotmail.com](mailto:zhao_jian_hua@hotmail.com)

Tel: +86-10-64805349

ORCID ID: 0000-0003-2554-1930 (J.-H.Z.)


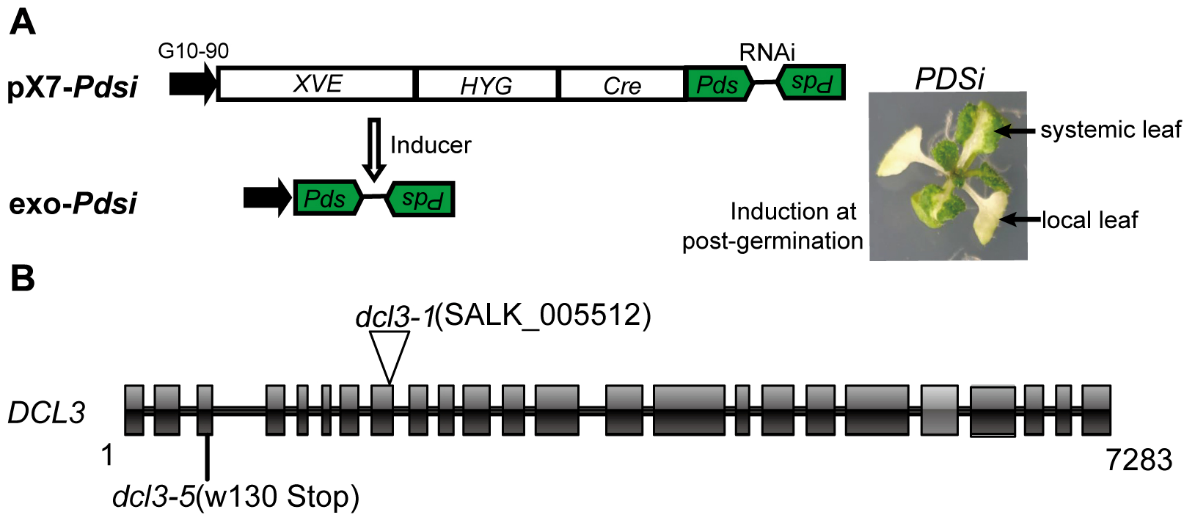


**Fig. S1 Schematic diagrams of the chemical-inducible CLX recombination system and dcl3 mutants.**

**(A)** Schematic of the structural features of the primary transgene (*pX7-Pdsi*) and the exo-*Pdsi* after Cre/loxP recombination, as well as the phenotype of induced *PDS* silencing at the postgermination stage (one-week-old seedlings were treated with inducer for one week). Induction of *PDS* silencing in local leaves (arrow) showing a uniform photobleaching phenotype. exo-*Pdsi*-produced siPds-induced endo-*PDS* silencing in systemic leaves showed a photobleaching phenotype limited to areas near the veins (arrowhead) (Guo et al. 2003).

**(B)** Schematic diagrams of the T-DNA insert mutant *dcl3-1* (SALK_005512) and the *dcl3-5* mutation position.


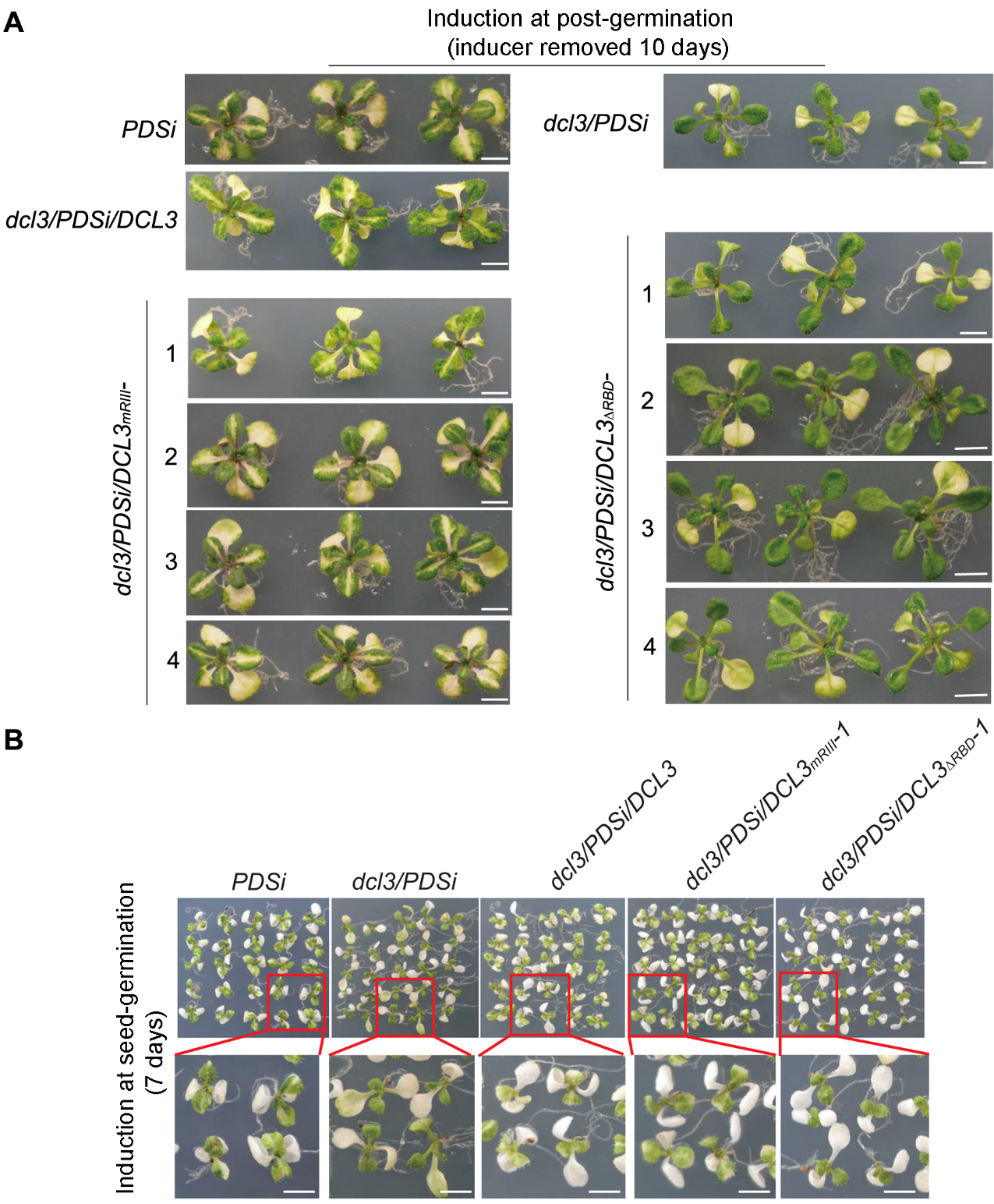


**Fig. S2 Phenotypes of transgenic *Arabidopsis* at the seed germination and postgermination stages.**

**(A)** Phenotypes of *PDS* silencing induced at the postgermination stage in *PDSi*, *dcl3/PDSi*, *dcl3/PDSi/DCL3, dcl3/PDSi/DCL3_mRIII_* lines and *dcl3/PDSi/DCl3_∆RBD_* lines. Bar = 0.5 cm.

**(B)** Phenotypes of *PDS* silencing induced at the seed germination stage in *PDSi*, *dcl3/PDSi*, *dcl3/PDSi/DCL3*, *dcl3/PDSi/DCL3_mRIII_-1* and *dcl3/PDSi/DCl3_∆RBD_-1*. Photographs were taken at 14 days postinduction. Bar = 0.5 cm.

**
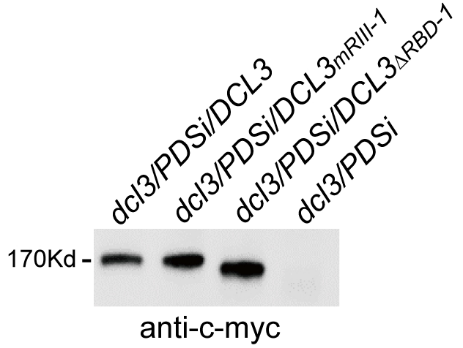
**

**Fig. S3 Expression of 6myc-DCL3 and its derivative mutants was confirmed by Western blotting using an anti-α-myc antibody.**

**
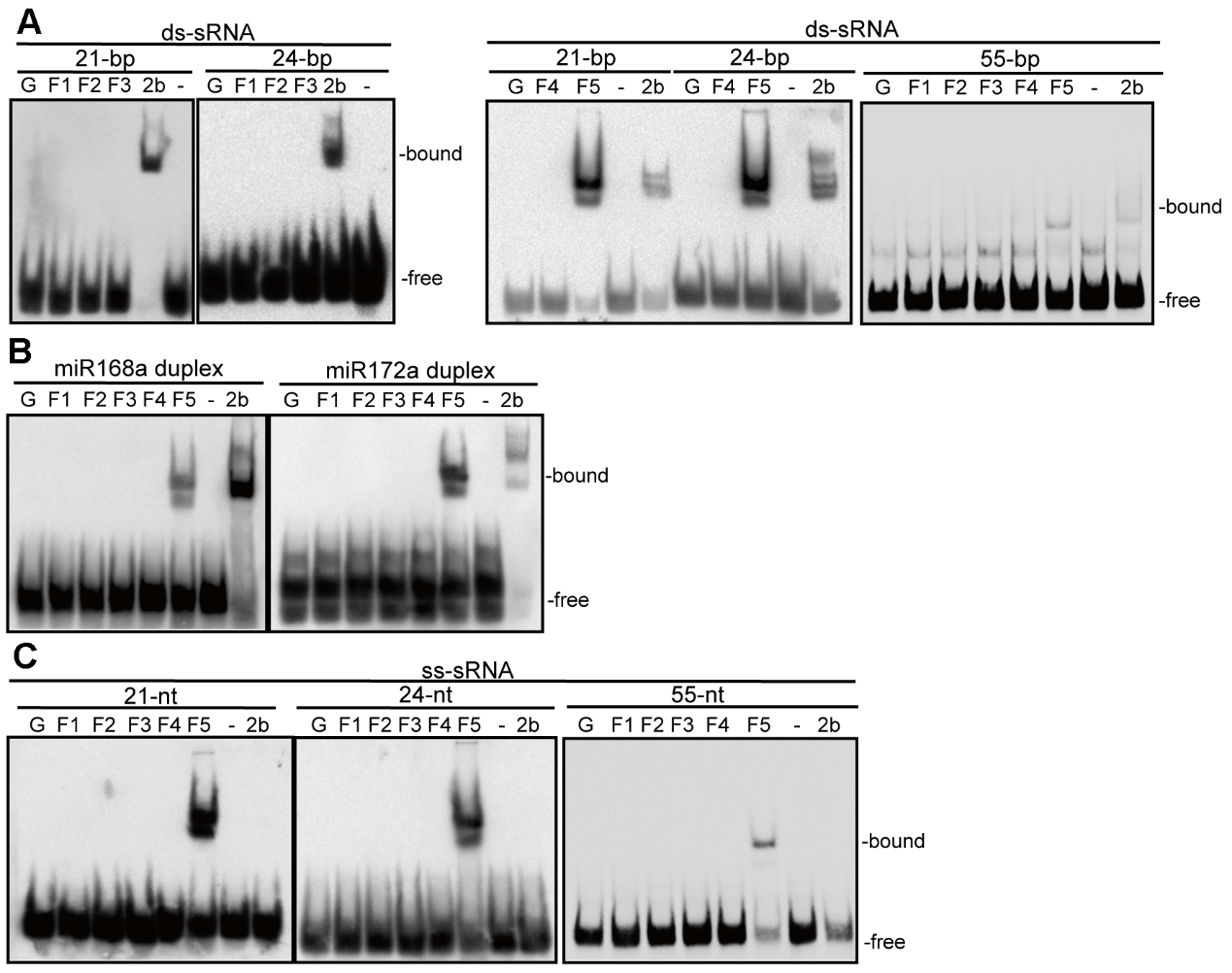
**

**Fig. S4 Detection of the RNA binding affinity of DCL3 by EMSA.**

**(A)** Gel mobility shift assay for detection of sRNA binding affinity of DCL3. Various GST-tagged F1, F2, F3, F4 and F5 were incubated with biotin-labeled synthetic 21-, 24- and 55-bp (base pair) ds-sRNA. GST-tagged SD2b and GST were used as controls. One nanomole of purified proteins was loaded in each assay. Bound and free probes are indicated.

**(B)** GST-tagged F1, F2, F3, F4 and F5 were incubated with biotin-labeled synthetic miRNA duplexes.

**(C)** GST-tagged F1, F2, F3, F4 and F5 were incubated with biotin-labeled 21-, 24-and 55-nt ss-sRNA. One nanomole of purified proteins was loaded in each assay.

**Table. S1 Primers and DNA oligos were used in this study.**

| **Primer name** | **Primer sequences(5’ to 3’)** | **Purpose** |
| --- | --- | --- |
| X-B-2b-F | TCTAGAGGATCCATGGAATTGAACGAAGGC | pGEX-4T-2-SD2b |
| S-X-2b-R | GAGCTCCTCGAGTCAGAAACGACCTTCCGC |  |
| DCL3-F1/F2-F | ATCTGGTTCCGCGTGGATCCATGCATTCGTCGTTGGAGCC | pGEX-4T-2-F1/-F2 |
| DCL3-F1-R | TCACGATGCGGCCGCTCGAGTTCAGGATTAAAAATCTTTG | pGEX-4T-2-F1 |
| DCL3-F2-R | TCACGATGCGGCCGCTCGAGAAGAAACATGATAGACTTAGA | pGEX-4T-2-F2 |
| DCL3-F3-F | ATCTGGTTCCGCGTGGATCCCAGGAAGTCTTACTAGACGA | pGEX-4T-2-F3 |
| DCL3-F3-R | TCACGATGCGGCCGCTCGAGATCTGGAGTTACAAGTGGAGAAA | pGEX-4T-2-F3 |
| DCL3-F4-F | ATCTGGTTCCGCGTGGATCCCATGAGTTTTGCAAGAAGCATAAACTTCAGCTTCCTCCATAC | pGEX-4T-2-F4 |
| DCL3-F5-F | ATCTGGTTCCGCGTGGATCCAAACTTCAGCTTCCTCCATACC | pGEX-4T-2-F5 |
| DCL3-F4/F5-R | TCACGATGCGGCCGCTCGAGCTTTTGTATTATGACGATCTTGC | pGEX-4T-2-F4/F5 |
| DCL3-F | TGTGAAGGAGATTGGATCCATGCATTCGTCGTTGGAGCC | pBI121-6myc-DCL3/-DCL3_∆RBD_/-DCL3_mRIII_ |
| DCL3-R1 | TGGGATAACCATGGATCCCTACTTTTGTATTATGACGATC |  |
| plocex-DCL3-F | TCGACCTGCAGGCGGCCGCACTAGTATGCATTCGTCGTTGGAGCCGGAG | plocex-DCL3-EGFP and  plocex-DCL3_mRIII_-EGFP |
| Plocex-DCL3-R | TCGCCCTTGCTCACCATCTCGAGCTTTTGTATTATGACGATCTTG | plocex-DCL3_∆RBD_-EGFP |
| DCL3-R2 | TGGGATAACCATGGATCCCTAATCTGGAGTTACAAGTGGAG | pBI121-6myc-DCL3_∆RBD_ |
| DCL3-P1 | TCATTTTCAATGGCGCGGTTGGCACTGCTCGGGGCTTCAGTCTTGAAG | RNaseIII-1 mutants |
| DCL3-P2 | CTTCAAGACTGAAGCCCCGAGCAGTGCCAACCGCGCCATTGAAAATGA | RNaseIII-1 mutants |
| DCL3-P3 | TCCTATTCATACGCGAGATTAGCGTTTCTTGGCGCTTCTGTACTGGAT | RNaseIII-2 mutants |
| DCL3-P4 | ATCCAGTACAGAAGCGCCAAGAAACGCTAATCTCGCGTATGAATAGGA | RNaseIII-2 mutants |
| PDS-F | TGAAGATGGGGACTGGTATGAG | PDS qRT-PCR |
| PDS-R | GTGCTGGTAGGACATCTGGGA |  |
| CMV-RNA3-F | TCCGAAGCCTTCAGACCGCAG | CMV qRT-PCR |
| CMV-RNA3-R | GCTGCTGCGACAAGACTCTA |  |
| siR1003 probe | ATGCCAAGTTTGGCCTCACGGTCT | siR1003 detection |
| U6 probe | GCTAATCTTCTCTGTATCGTTCC | U6 detection |
| AtRep2 probe | GCGGGACGGGTTTGGCAGGACGTTACTTAAT | AtRep2 detection |
| miR159 probe | TAGAGCTCCCTTCAATCCAAA | miR159 detection |

**Table. S2 RNA oligos were used for EMSA.**

| RNA oligos | Sequences(5’ to 3’) |  |
| --- | --- | --- |
| siRGFP-guide | 5’UCAACAGGAUCGAGCUUAAGG3’ | 21-nt ss |
| siRGFP-messenger | 5’UUAAGCUCGAUCCUGUUGACG3’ | 21-nt ss |
| ds siRGFP | 5’UCAACAGGAUCGAGCUUAAGG3’  3’GCAGUUGUCCUAGCUCGAAUU5’ | 21-bp, ds |
| miR168a guide | 5’UCGCUUGGUGCAGGUCGGGAA3’ | 21-nt ss |
| miR168a star | 5’CCCGCCUUGCAUCAACUGAAU3’ | 21-nt ss |
| miR168a duplex | 3’UUAAGCUCGAUCCUGUUGACG5’  5’UCGCUUGGUGCAGGUCGGGAA3’ | 21-bp, stem-loop |
| miR172a guide | 5’AGAAUCUUGAUGAUGCUGCAU3’ | 21-nt ss |
| miR172a star | 5’GUGGCAUCAUCAAGAUUCACA3’ | 21-nt ss |
| miR172a duplex | 5’AGAAUCUUGAUGAUGCUGCAU3’  3’ACACUUAGAACUACUACGGUG5’ | 21-bp, stem-loop |
| AtSN1-guide | 5’UGAAUUUCUGGUAUGGGUCCCGCC3’ | 24-nt ss |
| AtSN1-messenger | 5’CGGGACCCAUACCAGAAAUUCAUC3’ | 24-nt ss |
| dsATSN1 | 5’UGAAUUUCUGGUAUGGGUCCCGCC3’  3’CUACUUAAAGACCAUACCCAGGGC5’ | 24-bp, ds |
| 55-nt sense RNA | 5’UACAAGACACGUGCUGAAGUCAAGU  UUGAGGGAACACCCUCGUCAACAGGAUCG3’ | Long RNA binding affinity |
| 55-nt antisense RNA | 5’AUCCUGUUGACGAGGGUGUCUCCCU  CAAACUUGACUUCAGCACGUGUCUUGUAGU3’ |  |
